# Tailoring the Electron Pairing Process in a Pt–I Charge-Density-Wave Chain

**DOI:** 10.1021/jacs.6c09941

**Published:** 2026-07-08

**Authors:** Ying Luo, Ning Zhou, Yangbo Zhang, Ying-Fan Tan, Yuhui Yang, Xiong Wang, Qingyun Wan

**Affiliations:** † Department of Chemistry, 26451 The Chinese University of Hong Kong, Shatin, Hong Kong, Hong Kong SAR, China; ‡ Department of Physics, 25809 The University of Hong Kong, Pokfulam Road, Hong Kong, Hong Kong SAR, China; § Shanghai-Hong Kong Joint Laboratory in Chemical Synthesis, The Chinese University of Hong Kong, Shatin, Hong Kong, Hong Kong SAR, China

## Abstract

The electron pairing process in strongly correlated solid-state materials governs a range of important phenomena, such as superconductivity, charge density wave (CDW) orders, and so on. The ability to manipulate the electron pairs and charge orders is of central importance. Herein, we report the construction of a one-dimensional molecular superlattice structure featuring alternating Pt–I and Pt–Pt–I–I double-chains. The interchain electrostatic interactions could fundamentally reconfigure the electron pairing process, shifting it from a conventional localized mechanism to a delocalized, multisite pathway. This transformation stabilizes the first iodide-bridged Pt­(III) CDW chain. The delocalized electron pairing process within this CDW state elevates the material’s bulk conductivity by roughly 3 orders of magnitude compared to the localized state. Our findings establish a versatile chemical strategy for manipulating electron correlations and charge orders at the molecular level, providing a new design principle for strongly correlated electronic materials.

## Graphical abstract

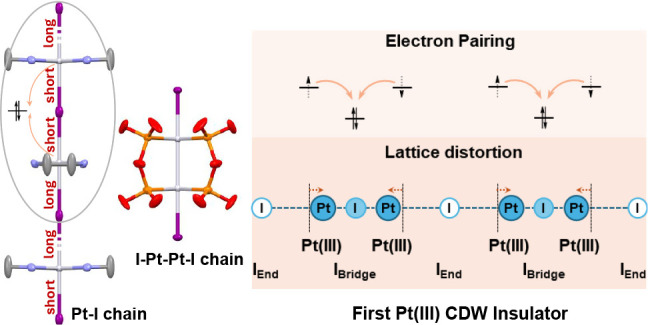

## Introduction

The rich phase diagram of strongly correlated electron materials—encompassing superconductivity, the Mott insulating state, and charge density wave (CDW) order—stems from electron–electron interactions and/or electron–phonon coupling.^1,2^ At the heart of these phases lies the electron pairing mechanism, which governs not only the formation of superconducting Cooper pairs but also the charge modulation and lattice distortion in CDW systems.^3–7^ Consequently, the study and control of the electron pairing process and mechanism hold significant importance across chemistry, physics, and materials science, driven by both fundamental and applied research objectives.^1–12^

One-dimensional (1D) systems, including molecular chains, nanowires, and surface atomic wires, offer a unique venue for exploring strong electron-correlation effects.^1,2,7,13–15^ In one dimension, lateral confinement forces electrons into strong interactions, amplifying correlation effects over single-particle band behavior. Such strong electron-correlation effects promote rich phase diagrams with competing orders.^1,2,7,14^

Our work directly manipulates the electron correlations and the electron pairing process by designing a tailored 1D environment—a molecular superlattice structure (Figure 1). Herein, a double-chain superlattice structure has been constructed using halogen-bridged transition-metal (MX) chain systems. One-dimensional MX chains are exemplary platforms for studying strong correlation effects.^8,12,16–28^ Among them, iodide-bridged platinum chains exhibit pronounced Peierls instability and robust CDW order.^14,26^ Conventionally, the ground state is described as a mixed-valence charge order alternating between Pt­(II) and Pt­(IV) sites in the MX chain, where electron pairing is confined to individual Pt orbitals, yielding strongly localized electronic states (Figure 1f).^14^ Although this localization stabilizes the CDW, it limits electronic mobility and the emergence of novel correlated behavior.

**Figure 1:**
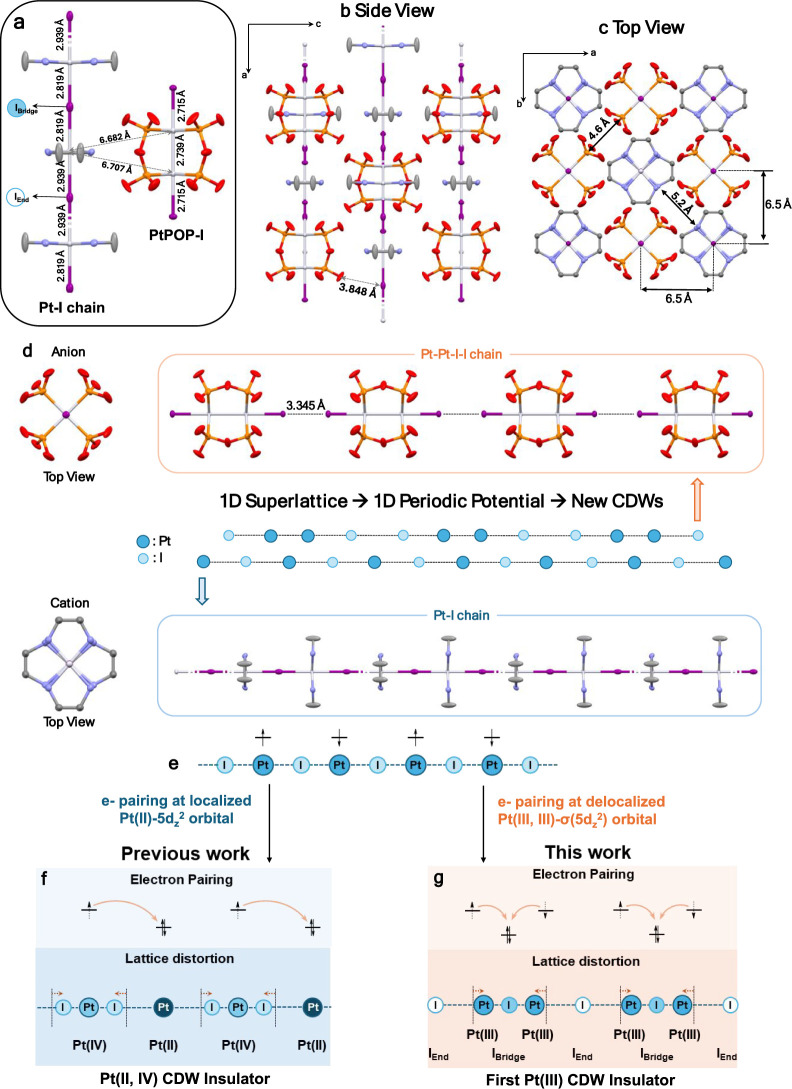
(a–d) Single crystal structures of the **[PtI­(en)**_**2**_ **]**_**2**_ **[PtPOP-I]** double-salt double-chain compound. Hydrogen atoms were omitted for clarity. Light gray atom: Pt; purple atom, I; dark gray atom, C; blue atom, N; red atom, O; orange atom, P. (e) Schematic drawing showing a one-dimensional Pt–I chain without electron pairing or lattice distortion. Charge orders, electron pairs, and lattice distortions formed in a (f) Pt­(II, IV) CDW insulator and (g) Pt­(III) CDW insulator.

Here, we employ a superlattice engineering approach to deliberately reconfigure the electron pairing pathway through tailored interchain coupling. This is realized in a double-salt, double-chain compound, **[PtI­(en)**_**2**_ **]**_**2**_ **[PtPOP-I]** (PtPOP-I = [Pt_2_(P_2_O_5_H_2_)_4_I_2_]^4–^, en = ethylenediamine), which comprises alternating ···Pt–I–Pt–I··· cationic MX chains and ···Pt–Pt–I–I–Pt–Pt–I–I··· anionic MMXX chains (Figure 1a–d). Interchain coupling shifts the ground state from a localized Pt­(II)/Pt­(IV) mixed-valence CDW state to a delocalized Pt­(III) CDW state in the MX chain, where the electron pairing process changes from a localized single-metal site to a delocalized multimetal site. This stabilizes a new type of charge order (Figure 1e–g), which is driven by an intense 1D periodic electric potential within the double-chain superlattice structure. Consequently, the delocalization enhances bulk conductivity significantly, establishing a direct link between the microscopic electron pairing mechanism and macroscopic electronic transport.

## Results and Discussion

The compounds were synthesized by slowly diffusing an aqueous solution of K_4_[Pt_2_^II,II^(P_2_O_5_H_2_)_4_] (**K**_**4**_ **PtPOP**) into a solution of **[Pt** ^**IV**^ **I**_**2**_ **(en)**_**2**_ **]­Cl**_**2**_. During this process, the I–Pt^IV^(en)_2_–I compound is reduced by **[PtPOP]** ^4–^ to form a ···–Pt–I–··· chain compound. Concurrently, **[PtPOP]** ^**4–**^ is oxidized to the Pt^III,III^ complex, **[PtPOP-I]** ^**4–**^, which acts as the counteranion. Single-crystal X-ray diffraction analysis confirmed the chemical structure as [PtI­(en)_2_]_2_[Pt_2_(P_2_O_5_H_2_)_4_I_2_], hereafter abbreviated as **[PtI­(en)**_**2**_ **]**_**2**_ **[PtPOP-I]**.

The crystal structure of the **[PtI­(en)**_**2**_ **]**_**2**_ **[PtPOP-I]** chain compound is displayed in Figure 1a–d. The planar [Pt­(en)_2_] units are bridged by two kinds of iodide ions (I^–^). One type (I_Bridge_) is situated at the midpoint between adjacent Pt centers, forming a shorter Pt–I bond of 2.819 Å, while the other type (I_End_) also resides at the midpoint but forms a longer Pt–I bond of 2.939 Å (Figure 1a). The alternation of these two bond lengths generates a linear extended Pt–I chain. The [Pt_2_(P_2_O_5_H_2_)_4_I_2_]^4–^ counteranion adopts a paddle-wheel structure featuring a Pt–Pt distance and a Pt–I distance of 2.739 and 2.715 Å, respectively. Close intermolecular contacts between the [Pt_2_(P_2_O_5_H_2_)_4_I_2_]^4–^ anions were identified, featuring an I–I distance of 3.345 Å (Figure 1d). Given that the van der Waals radius of iodine is 2.04 Å,^29^ this observed I–I distance is significantly shorter than the sum of the radii, indicating the presence of strong halogen–halogen interactions^30,31^ in this MMXX chain structure.

The oxidation state of Pt was assigned as +3 in both the anion and the cation. For the [Pt_2_(P_2_O_5_H_2_)_4_I_2_]^4–^ anion, this assignment is based on the presence of a direct Pt–Pt metal–metal bond and an overall – 4 charge. In the [PtI­(en)_2_]^2+^ cationic MX chain, although there are two different Pt–I distances: the Pt–I_Bridge_ and Pt–I_End_ distances, the iodide atom consistently occupies the midpoint between adjacent platinum atoms (Figure 1a). This contrasts sharply with conventional Pt­(II,IV) CDW chains, which feature iodide atom distortion between two Pt atoms.^14,26^ In Pt­(II,IV) CDW chains, the iodide atom displaces from the midpoint between the two Pt atoms, shifting closer to Pt­(IV) and farther from Pt­(II). This distortion produces alternating long Pt­(II)–I and short Pt­(IV)–I bonds. In the **[PtI­(en)**_**2**_ **]**_**2**_ **[PtPOP-I]** chain compound, the structural midpointing of iodide implies a single type of platinum site, supporting its assignment as Pt­(III). This I_End_–Pt–I_Bridge_–Pt–I_End_ repeating unit resembles the isolated [Pt_2_(en)_4_I_3_]­I_3_ molecule recently reported by Wakizaka, Yamashita, and co-workers.^32^ That molecule also features two distinct types of I atoms: a bridging iodide with a shorter Pt–I distance of 2.75066(14) Å and a terminal iodide with a longer distance of 2.9094(3) Å, with its Pt­(III) state assigned based on an iodide-bridged five-center ten-electron bond—a delocalized bond formed by Pt-5d_*z*2_ and I-5p orbitals. The MX chain reported herein can thus be viewed as an extended structure built from this [Pt_2_(en)_4_I_3_]­I_3_ building block, with each Pt atom adopting an average oxidation state of +3. Within this Pt–I chain, electron pairing occurs in the repeating I_End_–Pt–I_Bridge_–Pt–I_End_ units. This results in the formation of a delocalized bond among the involved Pt and I atoms. This bonding shortens the Pt–Pt distance and displaces the Pt atoms from the midpoint between the iodide atoms, resulting in a shorter Pt–I_Bridge_ distance relative to the Pt–I_End_ bonds (Figure 1g).

Stabilizing the Pt­(III) valence state, particularly in halide-bridged MX chain systems, has long been a significant challenge.^32^ The Pt­(III) state is conventionally realized only through direct Pt–Pt bonding.^33^ The underlying reason for this challenge can be rationalized and understood using the Mott-Hubbard model. In these MX chains, electron–phonon coupling (*S*), electron transfer (*t*), and on-site Coulomb repulsion (*U*) compete with each other.^34–36^ For typical MX chains based on Pt, a large *t* and a small *U* usually lead to a mixed-valence Pt­(II)/Pt­(IV) state accompanied by lattice distortion at I sites. In such cases, electron pairing is localized at Pt­(II) sites, and the distortion is driven by iodine displacements (Figure 1f). In contrast, the MX chain we report exhibits a distinct mechanism: electron pairing occurs between two Pt atoms within the −Pt–I_Bridge_–Pt– unit, and the concomitant lattice distortion is driven by the motion of these Pt atoms (Figure 1g). This unusual observation—a Pt-centered distortion coupled with an average Pt­(III) state—motivates further spectroscopic investigation to elucidate the detailed electronic structure of this compound.

Raman spectra were measured to obtain information about the charge orders in the **[PtI­(en)**_**2**_ **]**_**2**_ **[PtPOP-I]**.^17^ All Raman measurements were performed on single-crystal samples. Several crystals from different batches were measured to ensure reproducibility. The optical images of the **[PtI­(en)**_**2**_ **]**_**2**_ **[PtPOP-I]** single crystals are shown in Figure 2c. More optical and SEM (scanning electron microscopy) images of **[PtI­(en)**_**2**_ **]**_**2**_ **[PtPOP-I]** single crystals are summarized in Figures S1 and S2.

**Figure 2:**
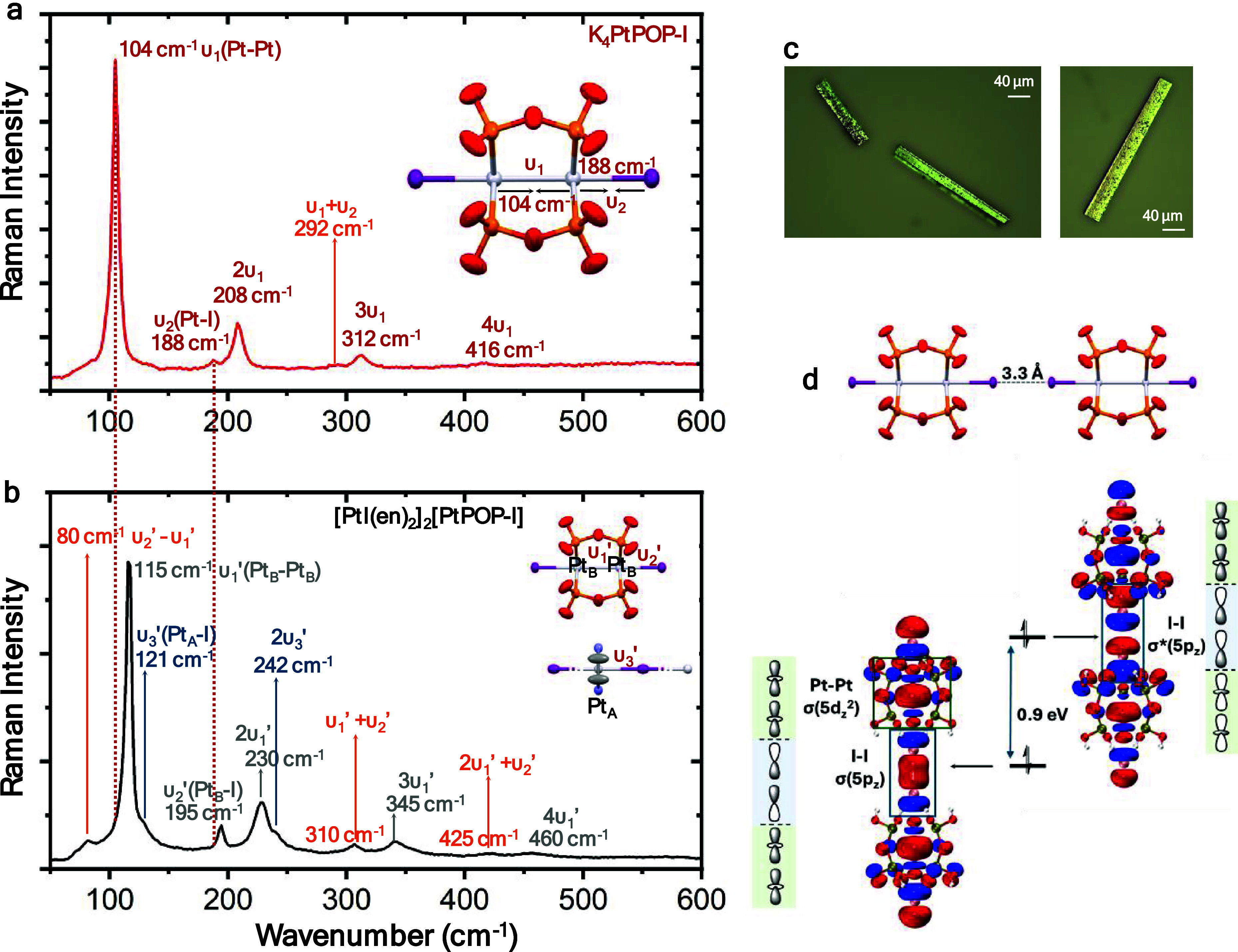
(a, b) Raman spectra of the single crystal sample for (a) **K**_**4**_ **PtPOP-I** and (b) **[PtI­(en)**_**2**_ **]**_**2**_ **[PtPOP-I]** at room temperature (λ_exc_ = 532 nm) in the air. (c) Optical images of the single crystals for compound **[PtI­(en)**_**2**_ **]**_**2**_ **[PtPOP-I]**. (d) Calculated MO (molecular orbital) diagram to show the I–I σ­(5p_*z*_) and σ*­(5p_*z*_) orbitals in the **[PtPOP-I]**_**2**_ structures based on the intermolecular I···I interaction.

Figure 2a,b shows the room-temperature Raman spectra of crystalline **K**_**4**_ **PtPOP-I** and **[PtI­(en)**_**2**_ **]**_**2**_ **[PtPOP-I]** samples, respectively. In Figure 2a, the strong band at 104 cm^–1^ is attributed to the Pt–Pt stretching mode, υ_1_(Pt–Pt), for **K**_**4**_ **[PtPOP-I]**.^37^ An overtone progression of υ_1_(Pt–Pt) up to the fourth order (208, 312, and 416 cm^–1^) is observed in this Raman spectrum. The presence of these overtones indicates strong electron–phonon coupling for Pt–Pt vibrations.^26^ A relatively weak band at ∼188 cm^–1^ is assigned to the symmetric Pt–I stretching mode in **K**_**4**_ **[PtPOP-I]**, in agreement with previously reported work.^37^ A weak peak at 292 cm^–1^ is also present in Figure 2a and is assigned to a combination band of the υ_1_(Pt–Pt) and υ_2_(Pt–I) modes (104 + 188 = 292 cm^–1^).

In the Raman spectrum measured for the **[PtI­(en)**_**2**_ **]**_**2**_ **[PtPOP-I]** compound, the Pt–Pt (υ_1_′) and Pt–I (υ_2_′) vibrational modes shift to 195 and 115 cm^–1^ in the MMXX chain respectively (Figure 2b). Moreover, the Pt–I vibration is more pronounced in the **[PtI­(en)**_**2**_ **]**_**2**_ **[PtPOP-I]** chain compound than in the **[PtPOP-I]** discrete molecule. An overtone progression of up to the fourth order (230, 345, and 460 cm^–1^) is observed for the Pt–Pt vibration mode. Three combination bands are observed in Figure 2b,^38^ assigned to υ_1_′(Pt_B_–Pt_B_) + υ_2_′(Pt_B_–I) at 310 cm^–1^, υ_2_′−υ_1_′ at 80 cm^–1^, and 2υ_1_′ + υ_2_′ at 425 cm^–1^.

The Pt–Pt and Pt–I distances are 2.739 and 2.715 Å in the MMXX chain of **[PtI­(en)**_**2**_ **]**_**2**_ **[PtPOP-I]** chain compound, compared to 2.754 and 2.746 Å in the discrete **K**_**4**_ **[PtPOP-I]** molecule.^39,40^ This bond shortening is attributed to intermolecular I···I interactions between adjacent **[PtPOP-I]** molecules. A short I···I contact (3.345 Å) observed in the crystal structure (Figure 1d) suggests significant I­(5p_*z*_)–I­(5p_*z*_) orbital overlap, which in turn draws the Pt–Pt and Pt–I atoms closer in the MMXX chain. To quantify the orbital overlap between two I-5p_*z*_ orbitals, we calculated the MO diagram for the **[PtPOP-I]**_**2**_ structures (Figure 2d). The resulting orbital splitting of 0.9 eV demonstrates substantial I···I interaction that facilitates moderate electron delocalization along the MMXX chain. Therefore, the enhanced Pt–I vibration and its Raman shift in the MMXX chain compound, compared to the discrete **K**_**4**_ **[PtPOP-I]** molecule, are proposed to result from this I···I halogen–halogen interaction.

Finally, aside from the Pt–Pt and Pt–I vibrations seen in the discrete **[PtPOP-I]** molecule, two broad peaks at 121 and 242 cm^–1^ are present in the Raman spectrum of Figure 2b. These two bands are assigned to υ_3_′(Pt_A_–I) and its overtone, 2υ_3_′, vibrational modes in the MX chain, respectively. In MX chain systems, only a CDW state with lattice distortion exhibits Raman-active vibrations for the Pt–I bond.^16^ The appearance of such peaks in Figure 2b suggests the existence of a CDW order in the MX chain, which is consistent with the observed crystal structures that show lattice distortion on the Pt sites. Notably, an active symmetric Pt–I Raman mode was also observed in the [Pt_2_(en)_4_I_3_]­I_3_ discrete molecule.^32^

Two-probe DC electrical conductivity measurements performed at room temperature on several single crystals of **[PtI­(en)**_**2**_ **]**_**2**_ **[PtPOP-I]** revealed non-ohmic behavior (Figure 3). The length, width, and thickness of the sample were measured from optical microscope images using a calibrated scale; the cross-sectional area was calculated accordingly. The non-ohmic *I*–*V* characteristics indicate that the carbon-paste contacts form Schottky barriers. Consequently, the measured two-probe resistance includes both the bulk sample resistance and the voltage-dependent contact resistances. To extract an approximate bulk resistivity, we performed an analysis of the region (10–20 V) where the *I*–*V* curve is nearly linear. This conductivity value of around 6.7 × 10^–6^ S cm^–1^ is indicative of semiconducting, rather than metallic, behavior. The conductivities of various Pt–I MX chains are summarized and compared in Table 1, which includes compounds with the same en ligand but different counteranions.^41^ As shown, the conductivity of **[PtI­(en)**_**2**_ **]**_**2**_ **[PtPOP-I]** is significantly higher—by 2–4 orders of magnitude—than those reported for **[PtI­(en)**_**2**_ **]**_**2**_ **Y**_**4**_ (where Y = I, ClO_4_, BF_4_). In contrast to the localized Pt sites in Pt­(II,IV) MX chains, electron pairing in our system occurs via a delocalized −Pt–I_Bridge_–Pt– orbital. This leads to a more delocalized electronic state in the **[PtI­(en)**_**2**_ **]**_**2**_ **[PtPOP-I]** MX chain, which enhances charge carrier mobility and improves the overall conductivity.

**Figure 3:**
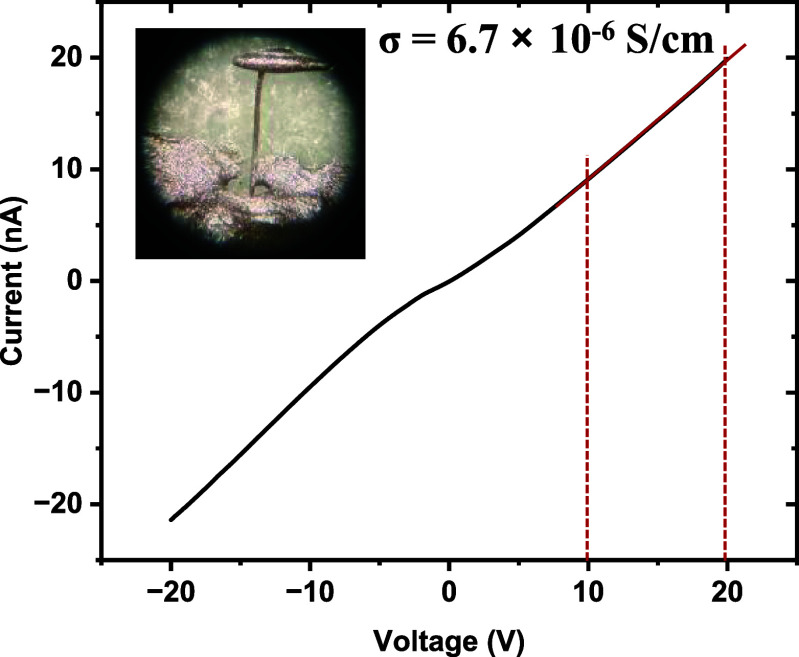
Two-probe electrical measurements for a single crystal of **[PtI­(en)**_**2**_ **]**_**2**_ **[PtPOP-I]** at room temperature. Inset picture shows the device structure. Carbon paste was used as electrodes. The resistivity was extracted from the linear (approximately ohmic) region of the *I*–*V* curve between 10 and 20 V.

**Table 1:** Summary of Conductivity Results Reported for MX Chain Single Crystals Measured with the Two-Probe Method at Room Temperature

**compound**	**conductivity/S cm** ^**–1**^	**ref**
**[Pt(en)**_**2**_ **] [PtI**_**2**_ **(en)**_**2**_ **]I**_**4**_	<10^–8^	^42^
**[Pt(en)**_**2**_ **] [PtI**_**2**_ **(en)**_**2**_ **](BF**_**4**_ **)**_**4**_	1.4 × 10^–9^	^43^
**[Pt(en)**_**2**_ **] [PtI**_**2**_ **(en)**_**2**_ **](ClO**_**4**_ **)**_**4**_	1.6 × 10^–8^	^43^
**[PtI(en)**_**2**_ **]**_**2**_ **[PtPOP-I]** (this work)	6.7 × 10^–6^	

We also fabricated several more devices with a two-probe geometry. The *I*–*V* curves from three independently prepared samples (voltage range 10–20 V) are summarized in Figure S4 in the Supporting Information. The curves show remarkably consistent resistance values, confirming the reproducibility and ruling out accidental contact effects. Single-crystal temperature-dependent resistivity measurements were further performed on the compound **[PtI­(en)**_**2**_ **]**_**2**_ **[PtPOP-I]** by using the two-probe method. The resistance (*R*) as a function of 1/*T* exhibits semiconductive behavior in Figure S11, as evidenced by a decrease in resistivity with an increase in temperature. The plot of ln­(*R*) versus 1/*T* (Figure S11b) shows a linear trend. Fitting the curve with a linear function yields an activation energy (*E*_a_) of 461 meV at ambient pressure, which corresponds to the energy difference between the transport level and the Fermi level of the compound **[PtI­(en)**_**2**_ **]**_**2**_ **[PtPOP-I]** and further supports the CDW energy gap.

The absorption spectrum of the powder **[PtI­(en)**_**2**_ **]**_**2**_ **[PtPOP-I]** chain compound was measured at room temperature (Figure 4), and compared with those of its building blocks, **K**_**4**_ **[PtPOP-I]** and **[PtI**_**2**_ **(en)**_**2**_ **]­Cl**_**2**_. An intense absorption band is observed at 736 nm (1.68 eV), which is absent in **K**_**4**_ **[PtPOP-I]** and **[PtI**_**2**_ **(en)**_**2**_ **]­Cl**_**2**_. This band is attributed to a valence band-to-conduction-band charge transfer transition within the compound’s MX chain structures. We also measured the optical absorption spectrum for **[PtI­(en)**_**2**_ **]**_**2**_ **[PtPOP-I]** in the low-energy range (400–2000 nm) using a conventional UV–vis–IR spectrometer on powder samples. As shown in Figure S5, no obvious absorption band is observed above 800 nm, and only the instrument background signal shows up. This *E*_CT_ energy is closely related to the Pt–Pt distance. The Pt–I chain compound reported in this work exhibits intermediate Pt–Pt distances of 5.638 and 5.877 Å, which are consistent with the observed *E*_CT_ energy of 1.68 eV.

**Figure 4:**
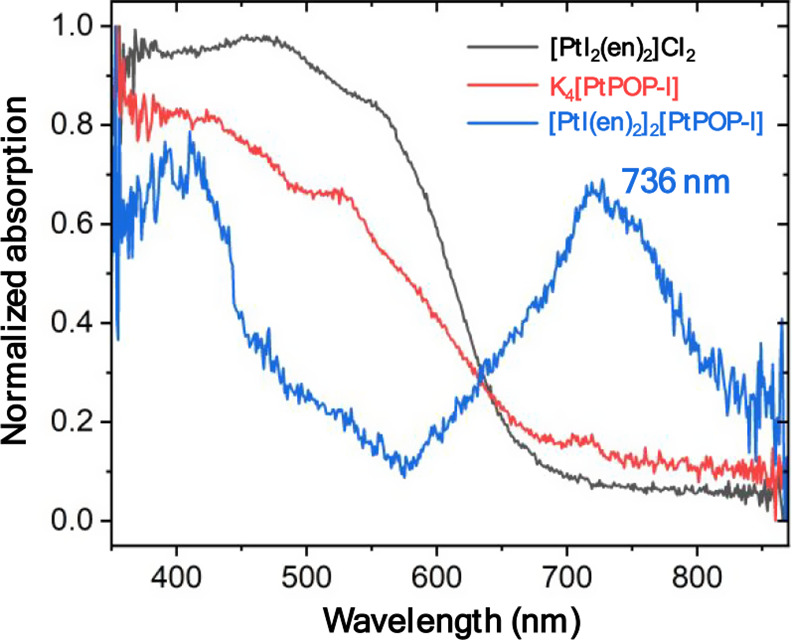
Absorption spectra of the powder sample for **K**_**4**_ **[PtPOP-I]**, **[PtI**_**2**_ **(en)**_**2**_ **]­Cl**_**2**_, and **[PtI­(en)**_**2**_ **]**_**2**_ **[PtPOP-I]** at room temperature.

To further assign the oxidation states of each the Pt atom in the **[PtI­(en)**_**2**_ **]**_**2**_ **[PtPOP-I]** compound, X-ray photoelectron spectroscopy (XPS) measurements were performed on this compound and its starting materials/building blocks, including **[Pt** ^**II**^ **(en)**_**2**_ **]­Cl**_**2**_, **[Pt** ^**IV**^ **I**_**2**_ **(en)**_**2**_ **]­Cl**_**2**_, **K**_**4**_ **PtPOP**, and **K**_**4**_ **PtPOP-I** compounds. The elemental composition of the **[PtI­(en)**_**2**_ **]**_**2**_ **[PtPOP-I]** chain compound was first analyzed via XPS (Figure 5f). The results confirm the presence of C, N, O, Pt, P, and I, which aligns with the chemical structure determined by X-ray crystallography.

**Figure 5:**
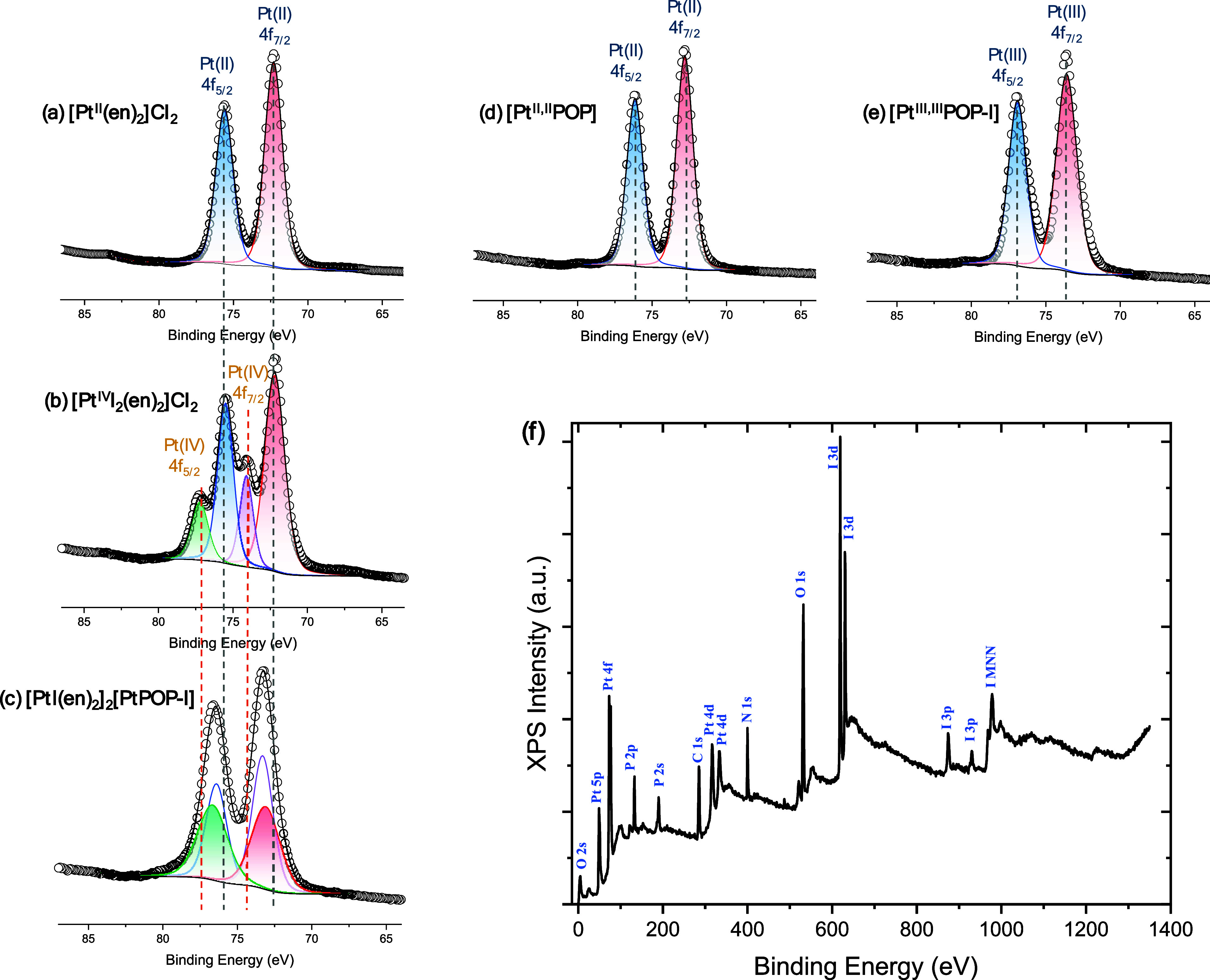
XPS spectra of the Pt 4f core levels for (a) **[Pt­(en)**_**2**_ **]­Cl**_**2**_, (b) **[PtI**_**2**_ **(en)**_**2**_ **]­Cl**_**2**_, (c) **[PtI­(en)**_**2**_ **]**_**2**_ **[PtPOP-I]**, (d) **K**_**4**_ **[PtPOP]**, and (e) **K**_**4**_ **[PtPOP-I]** compound. Black open circles: measured spectrum; black line: fitting curve; blue, red, purple, and green curves: components of the fitting curve. (f) Broad XPS scan of the sample **[PtI­(en)**_**2**_ **]**_**2**_ **[PtPOP-I]** compound.

Figure 5a–e present the high-resolution XPS spectra for the Pt 4f_5/2_ and 4f_7/2_ core levels. As shown in Figure 5a and the results summarized in Table 2, the Pt 4f spectrum of **[Pt­(en)**_**2**_ **]­Cl**_**2**_ exhibits two peaks at 72.3 eV (Pt^II^ 4f_7/2_) and 75.6 eV (Pt^II^ 4f_5/2_).^44^ In contrast, the Pt 4f spectrum of **[PtI**_**2**_ **(en)**_**2**_ **]­Cl**_**2**_ displays peaks at higher binding energies of 74.1 eV (Pt^IV^ 4f_7/2_) and 77.2 eV (Pt^IV^ 4f_5/2_), consistent with the expected increase in binding energy for higher oxidation states.^45^ Notably, the XPS spectrum of **[PtI**_**2**_ **(en)**_**2**_ **]­Cl**_**2**_ reveals partial reduction of Pt^IV^ to Pt^II^ on the sample surface under X-ray irradiation, as evidenced by the appearance of Pt^II^ 4f_5/2_ and Pt^II^ 4f_7/2_ peaks.^45^ This reduction from Pt^IV^ to Pt^II^ species has also been reported and observed in other Pt­(IV) complexes.^45^ The XPS spectrum of the **K**_**4**_ **[PtPOP]** compound shows two peaks at 72.8 and 76.2 eV, assigned to the Pt^II,II^ species (Figure 5d). For the **K**_**4**_ **[PtPOP-I]** compound, these peaks shift to higher binding energies of 73.6 and 76.9 eV, consistent with a higher Pt^III,III^ oxidation state.

**Table 2:** Binding Energies (eV) for Pt 4f Core Levels of **[Pt­(en)**_**2**_ **]­Cl**_**2**_, **[PtI**_**2**_ **(en)**_**2**_ **]­Cl**_**2**_, **[PtI­(en)**_**2**_ **]**_**2**_ **[PtPOP-I]**, **K**_**4**_ **[PtPOP-I]**, and **K**_**4**_ **[PtPOP]** Compound

compound	Pt 4f_5/2_	Pt 4f_7/2_
**[Pt(en)**_**2**_ **]Cl**_**2**_	75.6	72.3
**[PtI**_**2**_ **(en)**_**2**_ **]Cl**_**2**_	75.5, 77.2	72.2, 74.1
**[PtI(en)**_**2**_ **]**_**2**_ **[PtPOP-I]**	76.4, 76.7	73.1, 73.3
**K**_**4**_ **PtPOP**	76.2	72.8
**K**_**4**_ **PtPOP-I**	76.9	73.6

These spectra are further compared with those of the **[PtI­(en)**_**2**_ **]**_**2**_ **[PtPOP-I]** chain compound. In contrast to Figures[Fig fig5]a and b, no distinct Pt^II^ or Pt^IV^ peaks are observed in Figure 5c, indicating the presence of an average Pt^III^ oxidation state rather than a mixed-valence Pt^II,IV^ state in the chain structures. The Pt 4f spectrum of the **[PtI­(en)**_**2**_ **]**_**2**_ **[PtPOP-I]** compound was fitted with four peaks. The first set of peaks at 76.7 eV (4f_5/2_) and 73.3 eV (4f_7/2_) is attributed to the Pt atoms in the **PtPOP-I** anions. The second set, at 76.4 eV (4f_5/2_) and 73.1 eV (4f_7/2_), is assigned to the Pt atoms in the cationic MX chain. The slight shift of the **PtPOP-I** anion peaks to a lower binding energy, compared to the isolated **K**_**4**_ **PtPOP-I** compound, is attributed to the destabilization of the Pt orbitals caused by I···I halogen interactions and orbital overlap within the MMXX chain (Figure 2d). Both peaks are consistent with the Pt^III^ oxidation state in the **[PtI­(en)**_**2**_ **]**_**2**_ **[PtPOP-I]** compound.

Based on the assignment of the Pt^III^ oxidation state within the Pt–I chain, we measured the magnetic susceptibility of the **[PtI­(en)**_**2**_ **]**_**2**_ **[PtPOP-I]** compound to probe for the presence or absence of isolated unpaired spins. The compound exhibited small, negative magnetic susceptibility values across the entire measured temperature range (1.8–300 K), confirming its diamagnetic character and the absence of significant unpaired electron spins (Figure S6). This diamagnetic behavior is also observed in the [Pt_2_(en)_4_I_3_]­I_3_ discrete molecule.^32^ In both this compound and the Pt­(III) CDW chain reported herein, the diamagnetism is attributed to strong antiferromagnetic coupling between two Pt­(III) centers, mediated by robust orbital overlap interactions via the Pt-d_*z*2_ and I-p_*z*_ orbitals.^32^ Notably, our compound is not a collection of isolated single Pt­(III) ions; it is a one-dimensional Pt–I chain where two neighboring Pt atoms are bridged by an I atom. This structural dimerization leads to strong antiferromagnetic coupling between adjacent Pt­(III) centers via Pt-d_*z*2_ and I-p_*z*_ orbital overlap, resulting in a spin-singlet ground state (total spin *S* = 0) and, therefore, diamagnetic behavior.

### DFT and TDDFT Calculations

To further investigate the role of the **[PtPOP-I]** anion on the charge orders, we computationally compared the **[PtI­(en)**_**2**_ **]**_**2**_ **[PtPOP-I]** with a model system, **[PtI­(en)**_**2**_ **]**_**2**_ **I**_**4**_, where each **[PtPOP-I]** ^**4–**^ anion is replaced by four I^–^ anions. For the cationic chain, we selected Pt_4_I_5_ (consisting of two I_End_–Pt–I_Bridge_–Pt–I_End_ units) as our model compound. This choice was guided by the agreement between the calculated absorption spectrum for a Pt–I chain of this length and the corresponding experimental results (Figure S10). We calculated and compared the electrostatic potential (ESP) maps of both structures. As shown in Figure 6a, the **PtPOP-I** anions exhibit regions of strong negative charge (red regions on the O atoms) and are located on the same plane as the −Pt­(en)_2_– unit. We propose that the negative charges of **PtPOP-I** generate a strong electric field, which hinders on-site electron pairing in the Pt atomic orbitals on the in-plane single −Pt­(en)_2_– unit along the MX chain.

**Figure 6:**
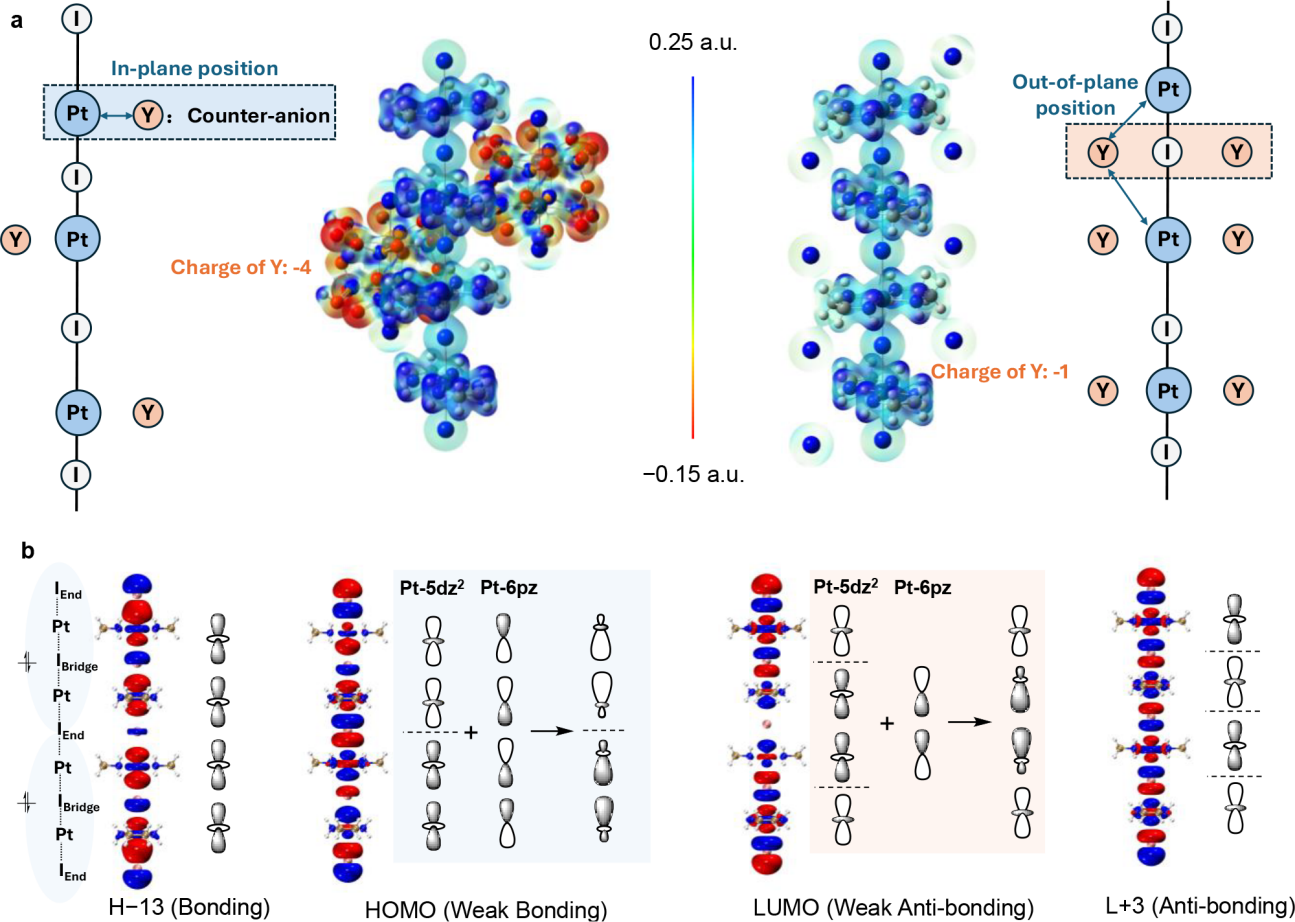
(a) Calculated ESP (electrostatic potential) maps of the Pt_4_I_5_ chain structure by changing the counter-anion (I or **PtPOP-I**). Y: counter-anion. Red region: negative charge and blue region: positive charge. (b) Calculated MO (molecular orbital) to show the Pt–Pt bonding, weakly bonding, weakly antibonding, and antibonding interaction for the **[PtI­(en)**_**2**_ **]**_**2**_ **[PtPOP-I]** chain compound, chain length: Pt_4_I_5_.

Compared to the **[PtPOP-I]** ^**4–**^ anion, the I^–^ anion possesses a lower negative charge and consequently induces a weaker electric field, as evidenced by the ESP map in Figure 6a. This results in a weaker electrostatic repulsion between the I^–^ anions and the Pt–I chain relative to the case involving the **[PtPOP-I]** ^**4–**^ anion. Another critical distinction lies in the spatial arrangement. In most reported MX chain systems with anions such as ReO_4_,^46^ HSO_4_,^47^ ClO_4_,^48–51^ (HPO_4_)­(H_2_PO_4_)­I,^52^ [CH_3_(CH_2_)_7_SO_3_],^53^ I,^12^ the counteranion is in the same plane as the bridging halogen atom, instead of the −Pt­(en)_2_– unit (right in Figure 6a). We consider that both the charge and location of the counteranion play important roles in regulating the electron pairing process in **[PtI­(en)**_**2**_ **]**_**2**_ **[PtPOP-I]**. The high charge of the **[PtPOP-I]** ^**4–**^ anion creates an intense 1D periodic electric potential within the superlattice. Furthermore, because it is coplanar with the −Pt­(en)_2_– units, this electric potential exerts strong electrostatic repulsion directly on the Pt sites. This combined effect effectively prevents electron localization and on-site pairing at any Pt center.

The electron pairing mechanism was further elucidated through MO calculations for a Pt_4_I_5_ model chain structure. As shown in Figure 6b, four key orbitals related to the Pt···Pt interaction were identified: a bonding orbital (H–13), a weakly bonding orbital (HOMO), a weakly antibonding orbital (LUMO), and a fully antibonding orbital (L+3). These orbitals are characterized by zero, one, two, and three nodal planes between neighboring Pt atoms, respectively. Strong orbital overlap between adjacent Pt and I atoms is also observed, indicating the formation of robust bonding interactions via the Pt–I bonds.^32^ A delocalized chemical bond forms among the Pt and I atoms because the bonding orbitals (H–13 and HOMO) are occupied while the antibonding orbitals (LUMO and L+3) are unoccupied. Consequently, in this Pt–I chain, where lattice distortion is induced by Pt atom movement, electron pairing occurs within these delocalized Pt–I_Bridge_–Pt bonding orbitals, leading to Pt­(III) charge density wave (CDW) chains. This contrasts with previously reported Pt–I chains, where distortion is driven by bridging I atoms and electron pairing is localized on single Pt sites, resulting in mixed-valence Pt­(II,IV) CDW chains.^8,16–28^

Notably, significant 5d–6p orbital hybridization on the Pt atoms is observed in both the HOMO and LUMO (Figure 6b). The lobe of the Pt 5d_*z*_^2^ orbital is strongly polarized through hybridization with the empty 6pz orbital. The 5d–6p hybridization serves to strengthen bonding and suppress antibonding interactions between the Pt atoms linked by the I_Bridge_ atom. The bonding interaction between the two Pt atoms is important to drive the lattice distortion centered on the Pt atoms in a Pt­(III) CDW chain system. This 5d–6p hybridization enhances the orbital overlap and overall bonding interaction between these two Pt atoms along the MX chain, thereby promoting electron pairing between the two Pt atoms rather than localization on a single Pt site.

Analysis of the structure, computational results, and spectroscopic properties of **[PtI­(en)**_**2**_ **]**_**2**_ **[PtPOP-I]** reveals a consistent electronic picture. The compound’s diamagnetism and average Pt­(III) oxidation state indicate a delocalized chemical bond formed among Pt and I atoms, where electron pairing occurs within the −Pt–I_Bridge_–Pt– unit. Concurrently, the presence of Raman-active Pt–I modes (Figure 2b) suggests a CDW insulating state. Lattice distortion usually accompanies the CDW state, which is further supported by the observed Pt-centered lattice distortion in its crystal structure. Computational results identified electrostatic interactions and counteranion locations as key factors governing charge orders and electron pairing mechanisms.

The MX chain system has long been regarded as a model system for investigating the interplay between e–ph and e–e interactions, relevant to high-*T*_c_ superconducting oxides.^14^ In this work, we have identified and verified the critical role of the counteranion in regulating charge order within the MX chain. We foresee potentially relevant insights for studying the charge orders in superconducting oxide systems.

The effect of cations (e.g., Y^3+^, Ba^2+^, and Ca^2+^) in cuprates has been extensively investigated for tuning superconducting properties through apical bond strength, chemical pressure, strain, and so on.^54^ Our study on MX chains now highlights a mechanism stemming from the counterion: a multiple in-plane charged counteranion can generate a strong local electric field. This field may effectively suppress electron localization and the formation of a mixed-valence state, thereby inducing a novel charge-order states.

## Conclusions

In summary, we have successfully synthesized and characterized a new iodide-bridged platinum chain compound that possesses a unique double-salt, double-chain 1D superlattice architecture. Through comprehensive experimental and computational investigations, we have identified a fundamentally new electron pairing mechanism in this system. Here, electrons form pairs in a delocalized orbital among multiple metal sites rather than on a single, localized metal site. This delocalization, driven by a strong electrostatic field inherent to the in-plane multiple charged anions, leads to significantly enhanced bulk conductivity. Our work establishes a versatile chemical strategy for manipulating electron pairing and CDW ordering in correlated systems.

## Supplementary Material



## Notes

Synthesis and characterization details, spectroscopic results, and computational details (PDF)
