# Supplementary material for: Tailoring the Electron Pairing Process in a Pt–I Charge-Density-Wave Chain
Source: J Am Chem Soc. 2026 Jul 8;148(28):30466–75. doi: 10.1021/jacs.6c09941 (PMC13397895; doi:10.1021/jacs.6c09941)
Supplement: Supplementary file 1 [file ja6c09941_si_001.pdf]

Supplementary information for

Tailoring the Electron Pairing Process in a Pt–I Charge-Density-Wave Chain.

Ying Luo,†[a] Ning Zhou,†[a] Yangbo Zhang,†[a] Ying-Fan Tan,[a] Yuhui Yang,[b] Xiong Wang[b] and Qingyun Wan*[a], [c]

Miss Y. Luo, Dr. N. Zhou, Mr. Y. Zhang, Mr. Y.-F. Tan, Prof. Dr. Q. Wan
[a] Department of Chemistry, The Chinese University of Hong Kong, Shatin, Hong Kong SAR (China)
E-mail: qingyunwan@cuhk.edu.hk

Miss Y. Yang, Dr. X. Wang
[b] Department of Physics, The University of Hong Kong Pokfulam Road, Hong Kong SAR (China)

Prof. Dr. Q. Wan
[c] Shanghai-Hong Kong Joint Laboratory in Chemical Synthesis, The Chinese University of Hong Kong, Shatin, Hong Kong SAR, China

Methods and Measurements

All starting materials were purchased from commercial sources and used as received. The solvents used for synthesis were of analytical grade unless stated otherwise.

X-ray crystal data were collected on a Bruker SMART CCD diffractometer and a Bruker KAPPA APEX II diffractometer with graphite-monochromatized Mo-Kα radiation (λ = 0.71073 Å) at 100 K. An empirical absorption correction was applied using the SADABS program.[1] The structures were solved by direct phase determination using the computer program SHELX-97 and refined by all-matrix least-squares with anisotropic thermal parameters for the non-hydrogen atoms.[2] Hydrogen atoms were introduced in their idealized positions and included in structure factor calculations with assigned isotropic temperature factors. Raman spectra were measured using a Horiba Scientific XploRA Plus spectrometer. Low laser power smaller than18 μW was applied to avoid potential sample damage. Two-probe electrical measurements were performed with a Keithley 4200-SCS parameter analyzer. Carbon paste electrodes were fabricated as follows: A small droplet of colloidal graphite paste was transferred onto each end faces of the sample using a needle under an optical microscope. A 0.02 mm diameter gold wire was immediately inserted into each paste droplet. The assembly was then air-dried for 3h to cure the paste. Absorption spectra were measured on powder samples using a PerkinElmer Lambda 950 UV-vis-NIR spectrometer. No polarizer was used because the samples were isotropic powders pressed into pellets, so light polarization is not applicable. Spectrometer calibration was performed daily using a standard white reference ($BaSO_4$). The same background subtraction and baseline correction procedures were applied to all spectra. X-ray photoelectron spectroscopy (XPS) spectra were acquired on a Thermo Scientific K-Alpha spectrometer. Temperature dependence χT was measured on Quantum Design Magnetic Properties Measurement System (MPMS). The X-ray powder diffraction (XPRD) pattern of **$[PtI(en)_2]_2[PtPOP\text{-}I]$** crystal was simulated using Mercury software. XPRD pattern of **$[PtI(en)_2]_2[PtPOP\text{-}I]$** powder was recorded using Rigaku MiniFlex600. Cyclic voltammetry (CV) measurements were performed using a CH Instruments CHI 620F electrochemical workstation. Thermogravimetric analysis (TGA) curve was performed on a Heson HS-TGA-101 analyzer. Temperature-dependent conductivity measurements were performed on a Quantum Design DynaCool PPMS (Physical Property Measurement System) using an ETO (Electrical Transport Option) module and a two-probe configuration. The measurement was performed under ambient pressure. Gold wires were used to attach the crystal, and carbon paste was used as the electrode.

Synthesis and Characterization

K_4PtPOP, **$[Pt(en)_2]Cl_2$** and **$[PtI_2(en)_2]Cl_2$** were prepared according to literature procedures.[3] **K_4PtPOP**: A 5 mL aqueous solution of K_2PtCl_4 (2 mmol, 0.8 g) was added to phosphorous acid (34 mmol, 2.8 g). The mixture was refluxed for 2 h, during which the color of the solution turned yellow. The resulting solution was evaporated to dryness in an oven at 110 °C, yielding a light greenish solid. This solid was washed with methanol and acetone to remove excess phosphorous acid, and then dissolved in approximately 10 mL of water. After filtration, methanol was slowly added to the filtrate, leading to the formation of microcrystals. The product was collected by filtration, washed with methanol and diethyl ether, and obtained as a light yellow-green solid (0.9 g, yield: 40%). **$[PtI_2(en)_2]Cl_2$**: A sample of **$[Pt(en)_2]Cl_2$** (1 mmol, 0.3 g) was dissolved in 10 mL water to give a colorless solution. An excess of I_2 in 10 mL ethanol was added dropwise slowly to this solution, leading to the formation of a red solid. After stirring for 0.5 h, the solid $[PtI_2(en)_2]Cl_2$ was collected by filtration and washed with ethanol and diethyl ether. (283 mg, yield: 50%). **$[PtI(en)_2]_2[PtPOP\text{-}I]$**: A 0.5 mL aqueous solution of **K_4PtPOP** (1 equiv., 1 mg) and a 0.5 mL aqueous solution of $[PtI_2(en)_2]Cl_2$ (2 equiv., 1 mg) were separately placed in two 5 mm tubes. Both tubes were then placed inside a 10 mL vial. Water was slowly added along the vial wall until the liquid level surpassed the tubes. The vial was sealed to allow for slow interdiffusion. After approximately 5 days, dark green needle-like crystals were observed inside the tubes. The needle-like crystals were collected by filtration and washed with water, ethanol and diethyl ether. The obtained microcrystals showed good agreement between their experimental X-ray powder diffraction pattern and the simulated pattern calculated from the single-crystal structure as showed in **Figure S7**.

Computational Details

The structures of Pt_2I_3, Pt_4I_5, Pt_6I_7, Pt_8I_9, and **$[PtI(en)_2]_2[PtPOP\text{-}I]$** used for DFT and TDDFT calculations were taken directly from X-ray crystal structures without geometry optimization. The structure of **$[PtI(en)_2]_2I_4$** was obtained by appropriately modifying the **$[PtI(en)_2]_2[PtPOP\text{-}I]$** structure based on the crystal structure of $[Pt(chxn)_2I]I_2$.[4] All DFT and TDDFT calculations were performed

with Gaussian 16, Revision C.02.[5] The PBE0 functional[6] together with Grimme's D3BJ dispersion correction[7] was employed for all calculations. The 6-31G(d) basis set[8] was used for light elements (H, C, N, O, P), while the Stuttgart/Dresden (SDD) relativistic effective core potential and its associated valence basis set[9] were applied for heavy elements (Pt, I) to account for scalar relativistic effects. Based on the TDDFT results, the calculated absorption spectra were generated using Multiwfn 3.8(dev) with a Gaussian broadening of 0.2 eV full width at half maximum (FWHM).[10] Excited state analyses were conducted through hole-electron analysis within Multiwfn, and the corresponding visualizations were generated using VMD 1.9.3.[11] Molecular orbital diagrams were plotted using Multiwfn and VMD. Electrostatic potential maps were produced with GaussView 6.1.[12]

Table S1. Crystal data and structure refinements for **$[PtI(en)_2]_2[PtPOP\text{-}I]$** (CCDC: 2517931).

	**$[PtI(en)_2]_2[PtPOP\text{-}I]$**
Empirical formula	$C_8H_{40}I_4N_8O_{20}P_8Pt_4$
Formula weight	2104.2
Temperature / K	100
Crystal system	Tetragonal
Space group	I-4m2
a / Å	13.1050(17)
b / Å	13.1050(17)
c / Å	11.515(2)
α / °	90
β / °	90
γ / °	90
Volume / Å^3	1977.6(6)
Z	2
ρ_{calc} g/cm^3	3.520
μ/mm^{-1}	17.629
F(000)	1880
Crystal size / mm^3	0.400×0.200×0.200
Radiation	MoKα (λ = 0.71073)
2θ range for data collection/°	2.198 to 28.249
Index ranges	$-17 \leq h \leq 17$, $-17 \leq k \leq 15$, $-15 \leq l \leq 15$
Reflections collected	19463
Independent reflections	1308 [R_{int} = 0.0578]
Data/restraints/parameters	1308/6/68
Goodness-of-fit on F^2	1.114
Final R indexes [R>=2σ(I)]	R1 = 0.0231, wR2 = 0.0593
Final R indexes [all data]	R1 = 0.0238, wR2 = 0.0597
Largest diff. peak/hole / eÅ^{-3}	1.111/−2.066

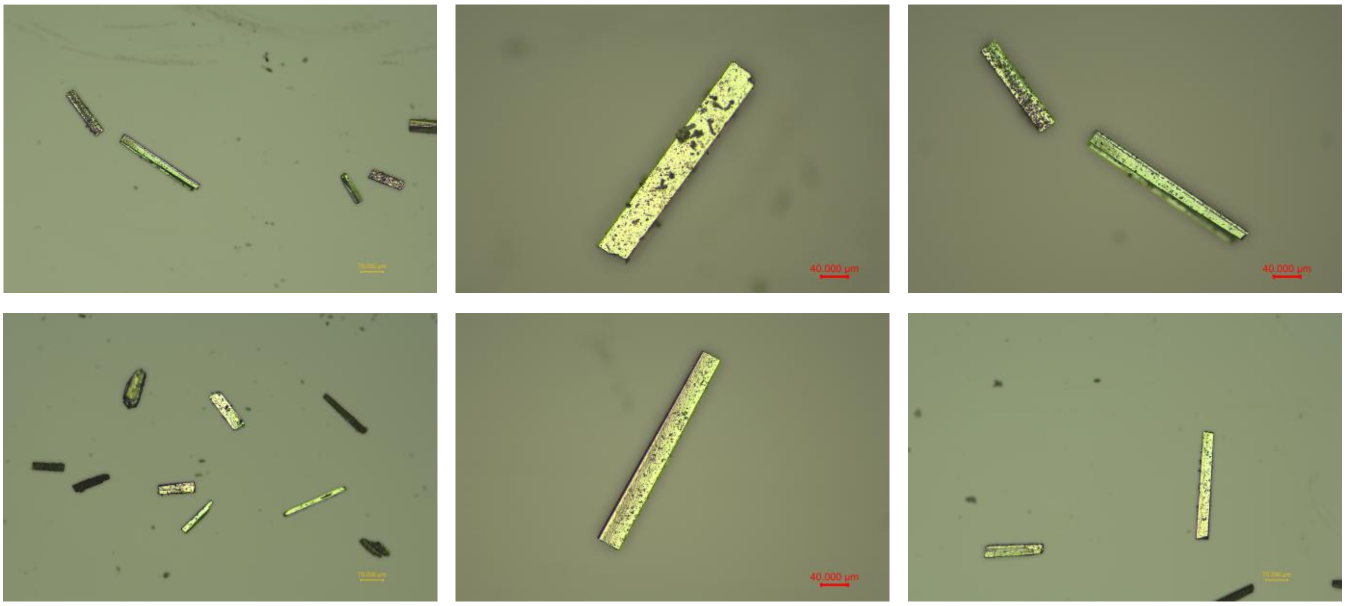

Figure S1. Optical images of the single crystals for compound **[PtI(en)$_2$]$_2$[PtPOP-I].**

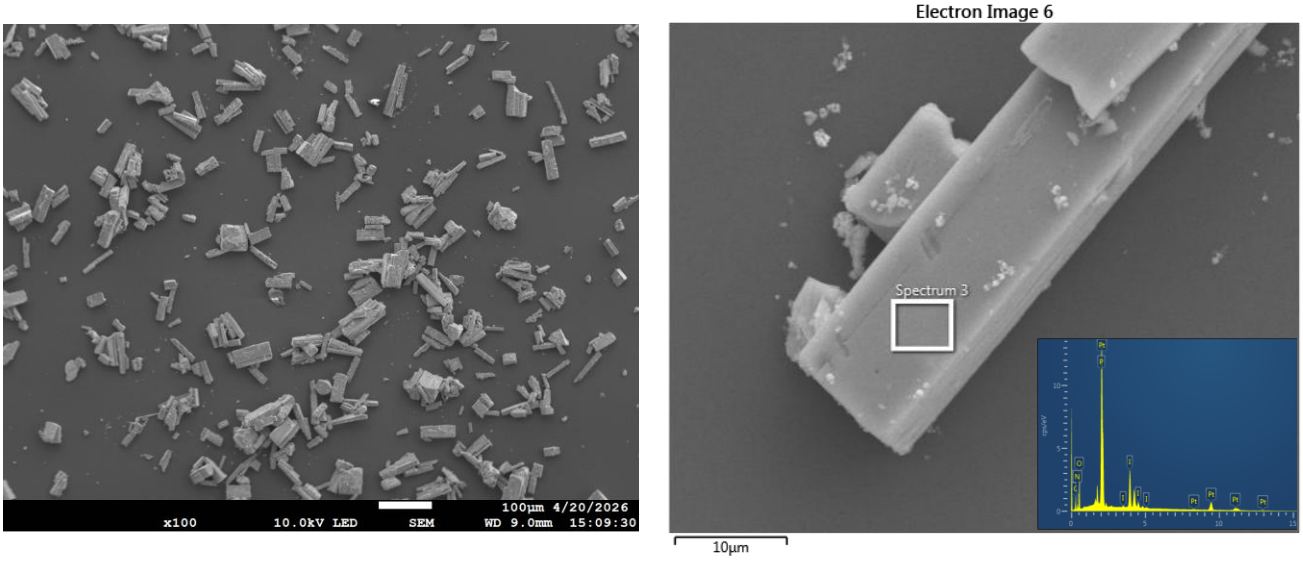

Figure S2. SEM images of the single crystals for compound **[PtI(en)$_2$]$_2$[PtPOP-I].** Inset: elemental analysis of a representative single crystal.

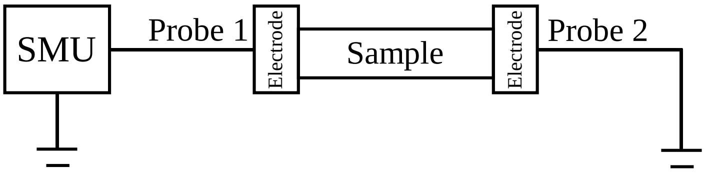

Figure S3. Measurement setup of a standard two-probe configuration with a source measure unit (Keithley 4200-SCS), electrode: carbon paste.

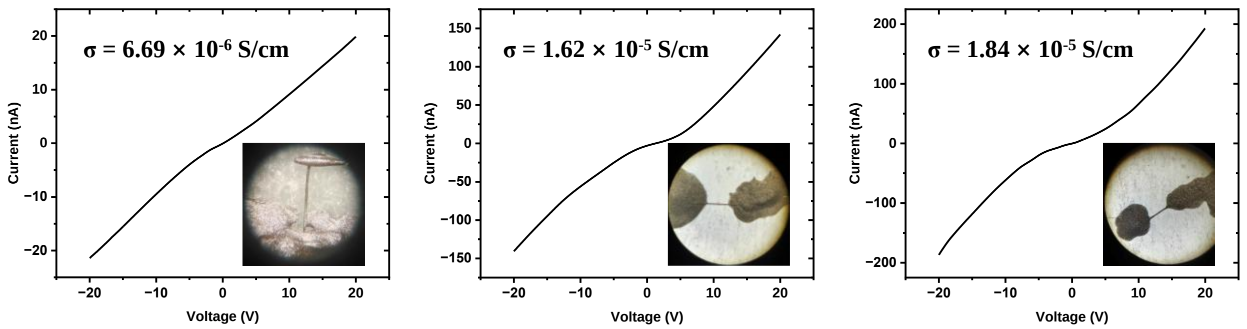

Figure S4. Two-probe I–V curves measured at room temperature on three single-crystal **$[PtI(en)_2]_2[PtPOP-I]$** samples, with the device structure illustrated in the inset. The resistivity was extracted from the linear (approximately ohmic) region of the I-V curve between 10 V and 20 V.

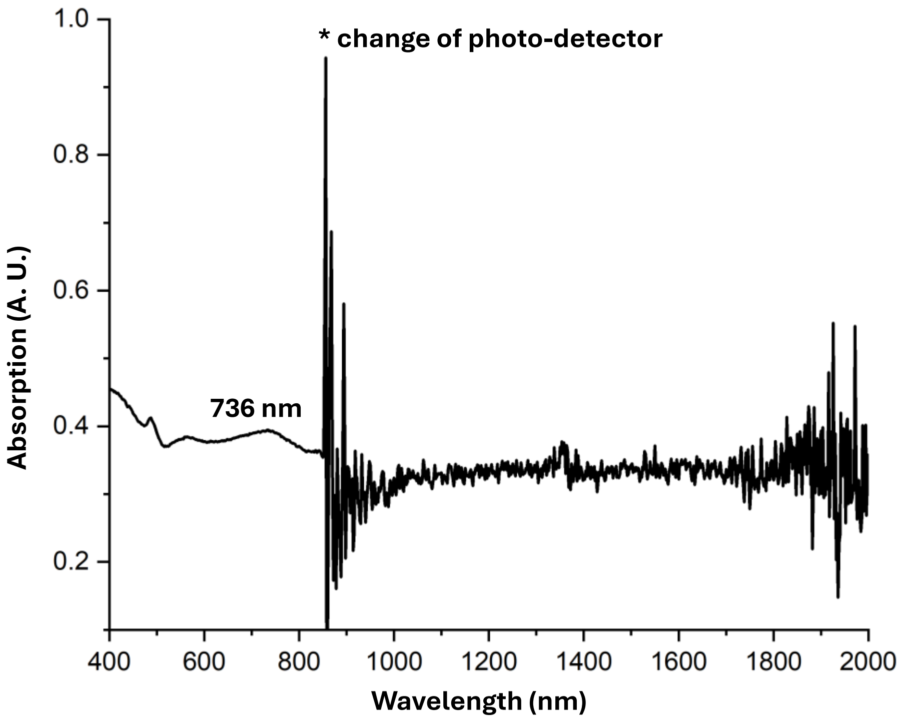

Figure S5. Absorption spectra of the powder sample for **$[PtI(en)_2]_2[PtPOP-I]$** at room temperature to show the low-energy range (900-2000 nm) features.

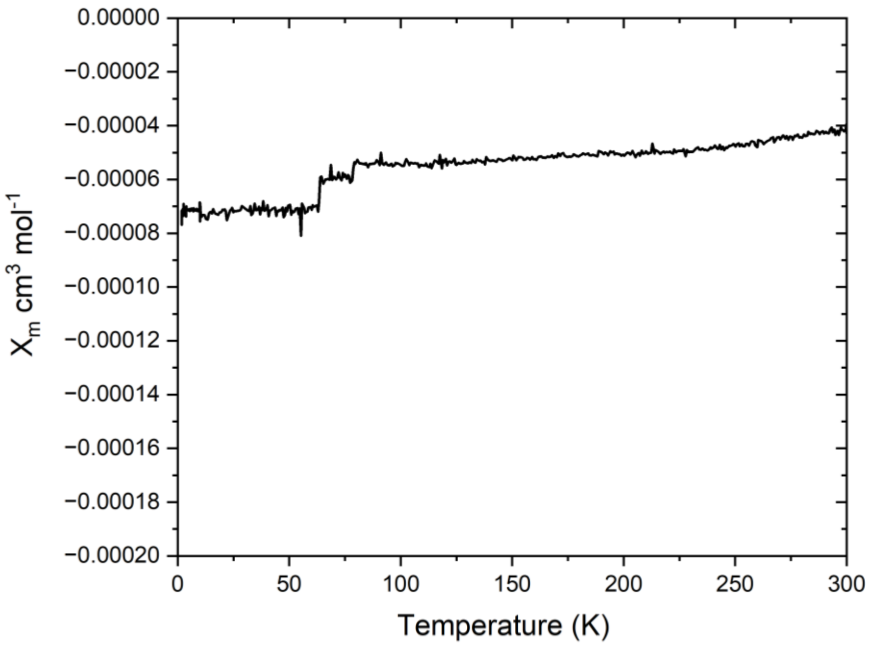

Figure S6 Temperature-dependent magnetic susceptibility (χ) of **$[PtI(en)_2]_2$[PtPOP-I]** under an applied field of 1000 Oe. The slight non-flatness of the curve and fluctuation arise from small sample displacements and background noise during the measurement.

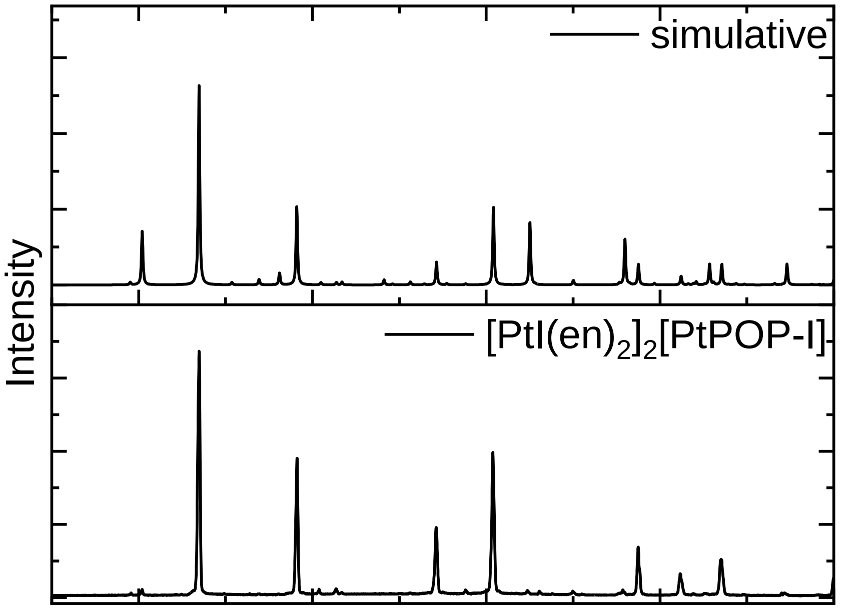

Figure S7. Comparison of experimental (bottom) and simulated (top) X-ray powder diffraction (XRPD) patterns for **$[PtI(en)_2]_2$[PtPOP-I]**.

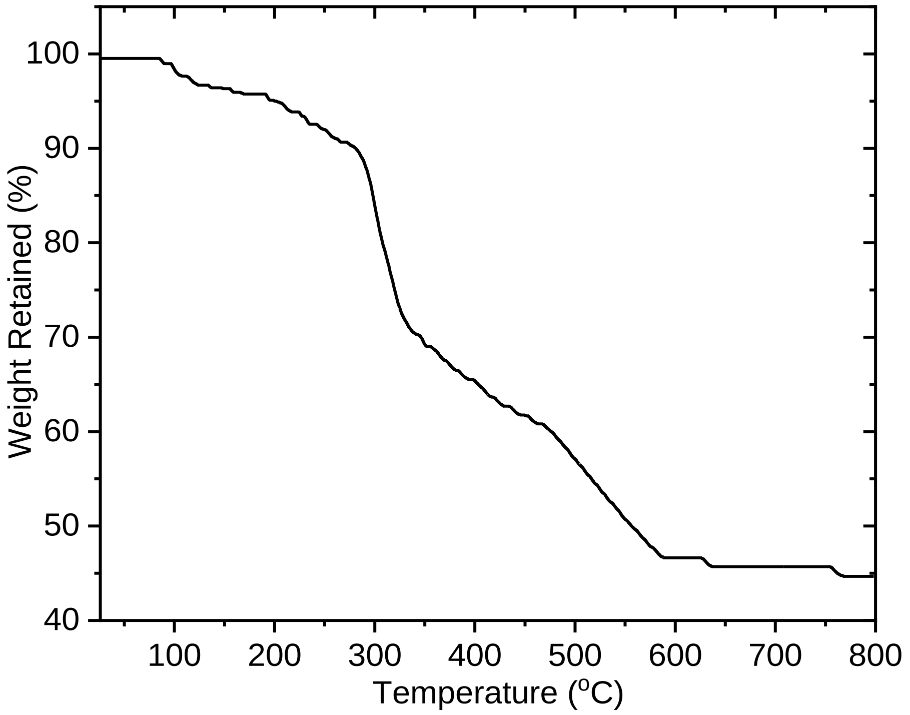

Figure S8. Thermogravimetric analysis (TGA) curve of **[PtI(en)$_2$]$_2$[PtPOP-I]**.

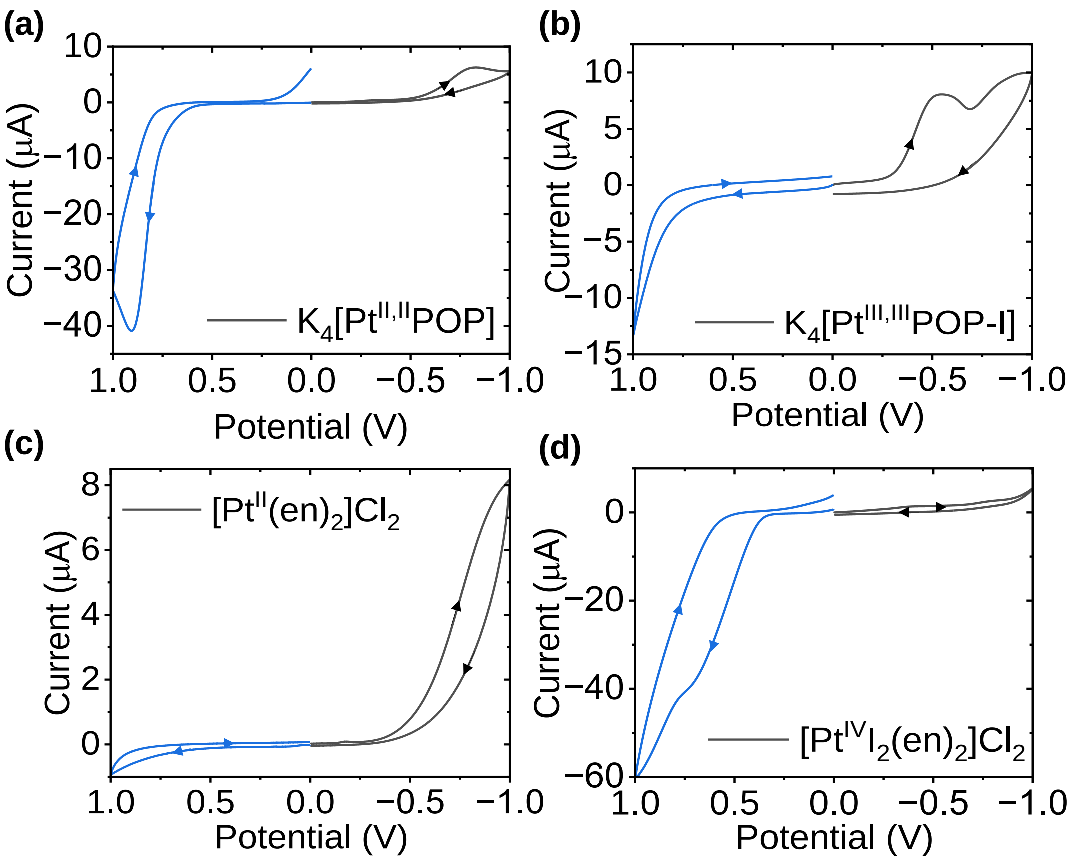

Figure S9. Cyclic voltammograms of a 1.0 mM aqueous solution of (a) **K$_4$[PtPOP]**, (b) **K$_4$[PtPOP-I]**, (c) **[Pt(en)$_2$]Cl$_2$** and (d) **[PtI$_2$(en)$_2$]Cl$_2$** containing 0.1 M TBACl as the supporting electrolyte. Working electrode: glassy carbon; reference electrode: Ag/AgCl; counter electrode: Pt wire.

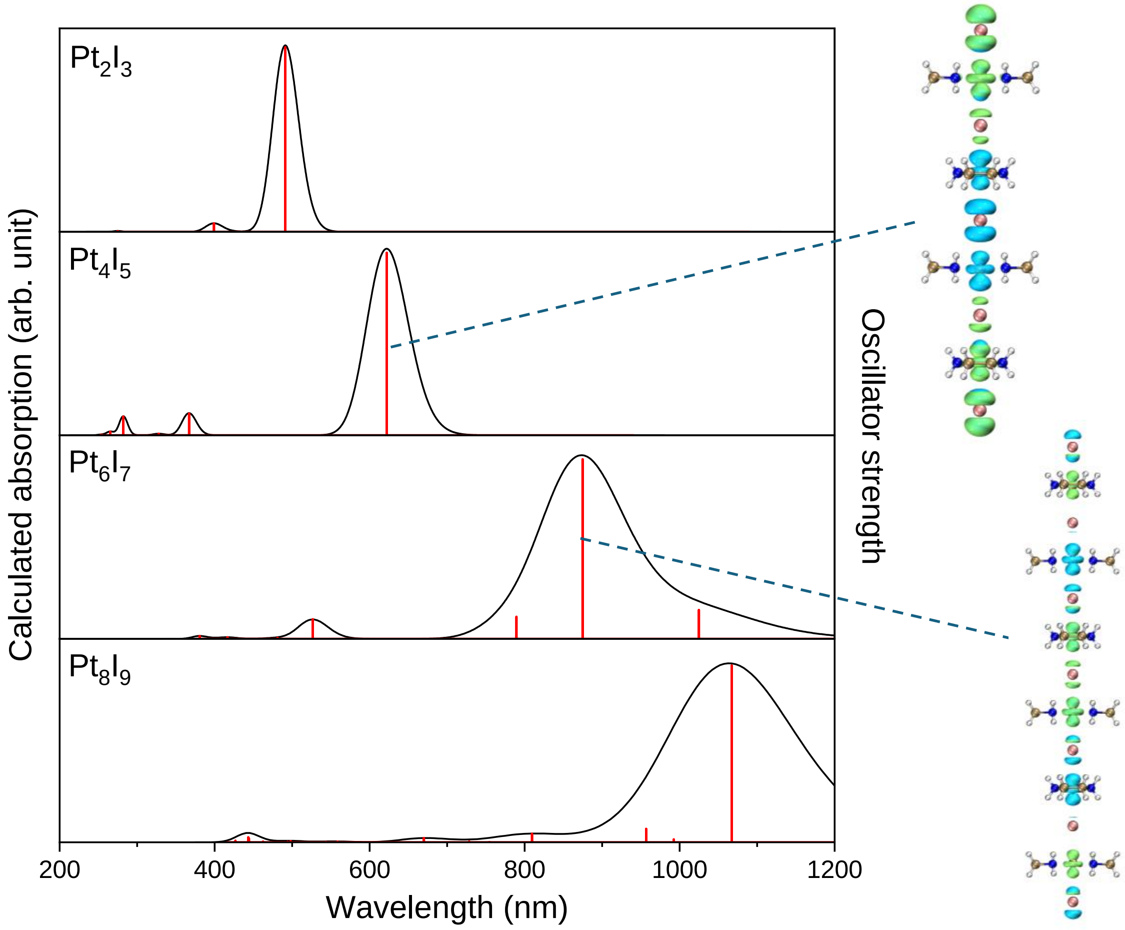

Figure S10. Calculated absorption spectra of Pt_2I_3, Pt_4I_5, Pt_6I_7 and Pt_8I_9 MX chains based on their X-ray crystal structures. Inset pictures show charge-transfer transition (isosurface = 0.002 a.u.), with electrons transferring from the blue region to the green region.

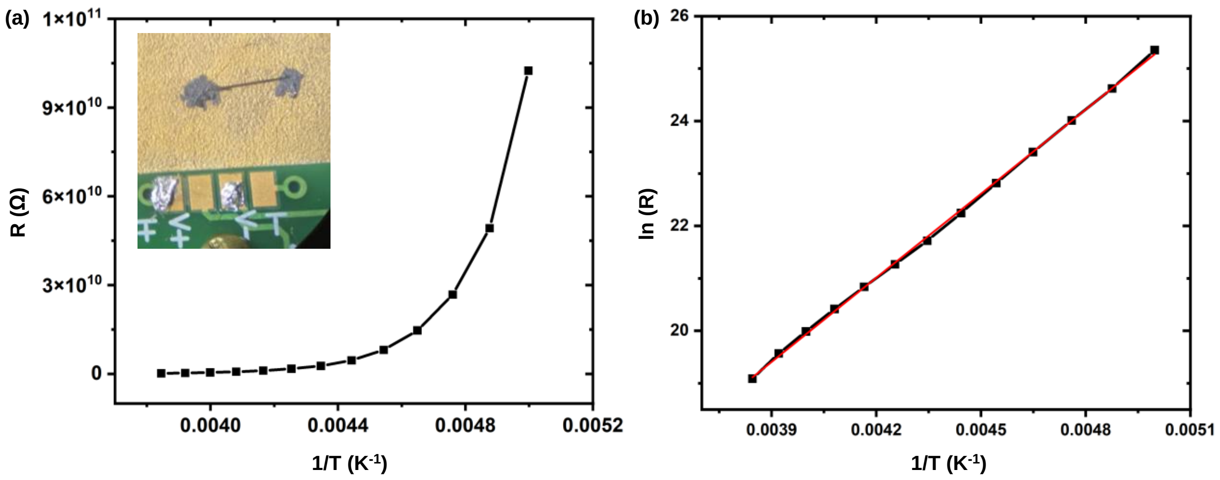

Figure S11. Temperature-dependent conductivity measurements on the single crystal sample of **[PtI(en)$_2$]$_2$[PtPOP-I]**. (a) Resistivity (R) as a function of 1/T. (b) Natural logarithm of resistivity [ln(R)] vs. 1/T, and its fitting curve to a linear function (red line). The inset in (a) shows the two-probe device structure. Details of device fabrication are provided in the part of Methods and Measurements in SI.

References:

(1) Sheldrick, G. M. Program for empirical absorption correction of area detector data. *Sadabs* **1996**.
(2) Sheldrick, G. SHELXTL Version 5.1. *Bruker AXS Inc., Madison, Wisconsin, USA* **1997**, *53719*.
(3) Che, C. M.; Butler, L. G.; Gray, H. B. Spectroscopic properties and redox chemistry of the phosphorescent excited state of $Pt_2(P_2O_5)_4H_8^{4-}$. *J. Am. Chem. Soc.* **1981**, *103* (26), 7796-7797. Nagai, Y.; Morikawa, M.-a.; Kimizuka, N. Near-infrared vapochromism in lipid-packaged mixed-valence coordination polymers. *Chem. Commun.* **2022**, *58* (13), 2112-2115.
(4) Takaishi, S.; Kawakami, D.; Yamashita, M.; Sasaki, M.; Kajiwara, T.; Miyasaka, H.; Sugiura, K.-i.; Wakabayashi, Y.; Sawa, H.; Matsuzaki, H.; et al. Dynamical Valence Fluctuation at the Charge−Density−Wave Phase Boundary in Iodide-Bridged Pt Compound $[Pt(chxn)_2I]I_2$. *J. Am. Chem. Soc.* **2006**, *128* (19), 6420-6425. DOI: 10.1021/ja060193b.
(5) *Gaussian 16, Revision C.02*; 2016. (accessed.
(6) Adamo, C.; Barone, V. Toward reliable density functional methods without adjustable parameters: The PBE0 model. *J. Chem. Phys.* **1999**, *110* (13), 6158-6170.
(7) Grimme, S.; Antony, J.; Ehrlich, S.; Krieg, H. A consistent and accurate ab initio parametrization of density functional dispersion correction (DFT-D) for the 94 elements H-Pu. *J. Chem. Phys.* **2010**, *132* (15). Grimme, S.; Ehrlich, S.; Goerigk, L. Effect of the damping function in dispersion corrected density functional theory. *J. Comput. Chem.* **2011**, *32* (7), 1456-1465.
(8) Hehre, W. J.; Ditchfield, R.; Pople, J. A. Self—Consistent Molecular Orbital Methods. XII. Further Extensions of Gaussian—Type Basis Sets for Use in Molecular Orbital Studies of Organic Molecules. *J. Chem. Phys.* **1972**, *56* (5), 2257-2261. DOI: 10.1063/1.1677527 (acccessed 8/9/2025). Hariharan, P. C.; Pople, J. A. The influence of polarization functions on molecular orbital hydrogenation energies. *Theor. Chim. Acta.* **1973**, *28* (3), 213-222. DOI: 10.1007/BF00533485.
(9) Igel-Mann, G.; Stoll, H.; Preuss, H. Pseudopotentials for main group elements (IIIa through VIIa). *Mol. Phys.* **1988**, *65* (6), 1321-1328. Andrae, D.; Häußermann, U.; Dolg, M.; Stoll, H.; Preuss, H. Energy-adjusted ab initio pseudopotentials for the second and third row transition elements. *Theor. Chim. Acta.* **1990**, *77*, 123-141.
(10) Lu, T.; Chen, F. Multiwfn: A multifunctional wavefunction analyzer. *J. Comput. Chem.* **2012**, *33* (5), 580-592. Lu, T. A comprehensive electron wavefunction analysis toolbox for chemists, Multiwfn. *J. Chem. Phys.* **2024**, *161* (8).
(11) Liu, Z.; Lu, T.; Chen, Q. An sp-hybridized all-carboatomic ring, cyclo[18]carbon: Electronic structure, electronic spectrum, and optical nonlinearity. *Carbon* **2020**, *165*, 461-467. DOI: https://doi.org/10.1016/j.carbon.2020.05.023. Humphrey, W.; Dalke, A.; Schulten, K. VMD: visual molecular dynamics. *J. Mol. Graph.* **1996**, *14* (1), 33-38.
(12) *GaussView, Version 6.1*; Semichem Inc.: Shawnee Mission, KS, 2016. (accessed.
